# Selective Carbonyl Reduction in Unsaturated Esters and Aldehydes by Transfer Hydrogenation

**DOI:** 10.1021/acs.organomet.5c00094

**Published:** 2025-07-17

**Authors:** Víctor Martínez-Agramunt, Lucas H. R. Passos, Dmitry G. Gusev, Eduardo Peris, Eduardo N. dos Santos

**Affiliations:** † Departamento de Química, 28114 Universidade Federal de Minas Gerais (UFMG). Av. Antônio Carlos 6627, 31270-901 Belo Horizonte, MG, Brazil; ‡ Institute of Advanced Materials (INAM), 16748 Universitat Jaume I (UJI). Av. Vicente Sos Baynat s/n, 12071, Castellón, CV, Spain; § 8431 Wilfrid Laurier University (WLU), Waterloo, Ontario N2L 3C5, Canada

## Abstract

Catalytic transfer hydrogenation (TH) is an alternative to the industrially relevant hydrogenation of carbonyl compounds, dismissing the use of pressurized reactors. Herein, we compare ruthenium and osmium pincer complexes as catalysts for the selective carbonyl reduction of the renewable methyl 10-undecenoate, myrtenal, and cinnamaldehyde. Their selective carbonyl reduction is challenging because they also have a C–C double bond susceptible to reduction or isomerization. The osmium complexes, used for the first time in TH, showed considerably better activity and selectivity than the ruthenium ones. The reactions were carried out at temperatures as low as 35 °C at short reaction times, and a solvent screening demonstrated that anisole, which has a high sustainability score, is also the most efficient solvent for these reactions. Finally, renewable ethanol was employed as a sacrificial hydrogen source, circumventing the use of the usually high-carbon-footprint dihydrogen.

## Graphical abstract

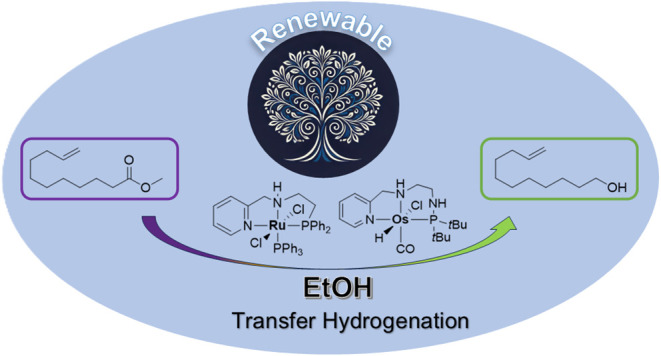

## Introduction

One of the most significant transformations in organic chemistry is the catalytic hydrogenation of unsaturated compounds,^1–5^ among which the reduction of esters and aldehydes to alcohols constitutes the basis for the syntheses of versatile building blocks for the industrial production of agrochemicals, pharmaceuticals, flavors, and fragrances.^6^ Catalytic hydrogenation has several advantages over the classical reduction methods that employ stoichiometric reactants (e.g., aluminum or boron hydrides) by reducing the amount of waste generated, simplifying the experimental workups, and allowing for a wider functional-group tolerance.^7^ For these reasons, catalytic hydrogenation has been recognized as a more environmentally benign synthetic method.^8^ For the homogeneously catalyzed reduction of esters and aldehydes, the two main approaches are the direct reaction with dihydrogen and transfer hydrogenation (TH), in which hydrogen is extruded from an alcohol or formic acid. Transfer hydrogenation has several advantages over hydrogenation under H_2_, such as (i) no pressurized reactors are required; (ii) the hydrogen sources can be renewable; and (iii) the coproduct can be recycled (e.g., isopropanol/acetone).^4,9–17^ Considering that conventional industrial hydrogen production generates ca. 6 tons of carbon dioxide per ton of hydrogen, its replacement with a lower-carbon-footprint alternative is of utmost importance for the sustainability of the process.

The mechanistic details of catalytic hydrogenation reactions can be found in the literature,^18–20^ but to understand the role of the hydrogen source, it is appropriate to briefly discuss the thermodynamics of the hydrogenation of carbonyl compounds. The Gibbs energy for ethanol dehydrogenation to give ethyl acetate is ca. 2 kcal per mole of H_2_ produced.^21^ This relatively low “cost” of H_2_ extrusion from ethanol makes the Gibbs energy of transfer hydrogenation of typical ketones in ethanol about −3.5 kcal/mol. Thus, transfer hydrogenation in ethanol is a thermodynamically favorable process, in contrast to the reduction of ketones in 2-propanol, which has the Gibbs energy near zero (Δ*G* ≈ 0). Esters, which are more challenging to reduce than ketones, are typically reduced under H_2_ ^22–24^ because the TH of esters in 2-propanol is an endergonic process. These thermodynamic considerations make ethanol a candidate for a hydrogen source for the reduction of esters because even if the TH of esters in ethanol can be expected to be thermoneutral, this source is inexpensive and can be used in excess, which can be easily recycled. It is important to point out that ethanol can be industrially obtained through low-carbon-footprint processes.^25–29^

TH is a well-established process to reduce ketones to alcohols. Less common is the use of TH for aldehyde reduction, and few examples are found for ester reduction, particularly the ones containing additional C–C double bonds.^30–33^ The difficulty in controlling the chemoselectivity for the hydrogenation of α,β-unsaturated aldehydes under mild conditions was reported by Wang and co-workers.^34^

In Figure 1, some pincer complexes active in ester hydrogenation are presented. **Ru-1**, **Ru-2**, and **Ru-3** are known to be efficient for the reduction of esters under hydrogen,^35^ and **Os-1**, **Os-2**, and **Os-3** demonstrated the efficiency in both catalytic hydrogenation of esters under H_2_ and in the acceptorless dehydrogenative coupling (ADC) of alcohols.^36^

**Figure 1:**
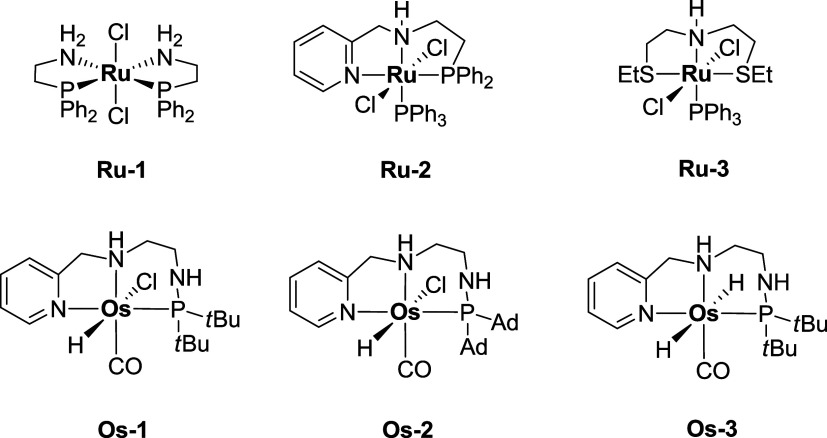
Ruthenium and Osmium Catalysts Used in This Work: **Ru-1** (PNNP), **Ru-2** (PNN), **Ru-3** (SNS), **Os-1** (H–Cl-tBu), **Os-2** (H–Cl-Ad), and **Os-3** (H–H-*t*-Bu).

In this work, we explored the catalytic activity of the complexes depicted in Figure 1 for the reduction of unsaturated esters and aldehydes by TH. This work is in line with the Green Chemistry principles^37^ in terms of using efficient catalytic transformations at low temperatures,^38^ using anisole as a solvent, which is among the most recommended solvents according to the GSK Solvent Guide,^39^ and using renewable ethanol, a feedstock with a low carbon footprint, as the hydrogen source.

## Results and Discussion

For the initial investigations, we used methyl 10-undecenoate (Scheme 1) as a substrate. This compound is a renewable precursor to polyamides, obtained by the pyrolysis of castor oil. Methyl 10-undecenoate (**1**) is a convenient substrate for testing the selectivity of reduction of the carbonyl group against the concurrent reduction or isomerization of the C=C bond. The target product for the selective ester reduction is 10-undecen-1-ol (**2**), and several side-reactions can occur (Scheme 1). Considering that the hydrogen source is a primary alcohol (ethanol) and a base (sodium methoxide) is employed as a cocatalyst, the transesterification products **5** and **6** are expected intermediates that can evolve to **2**. The reduction of the C=C bond in **1**, **2**, or **5** would lead to the saturated products **3**, **4**, or **6**, respectively. Intermediates **3** and **6** can ultimately evolve to **4**. The final alcohol products **2** and **4** can, in principle, be engaged on transesterification, resulting in esters analogous to **5** or **6**. Finally, the terminal C=C can be isomerized to form the positional isomers of **2** (not shown in Scheme 1).

**Scheme 1:**
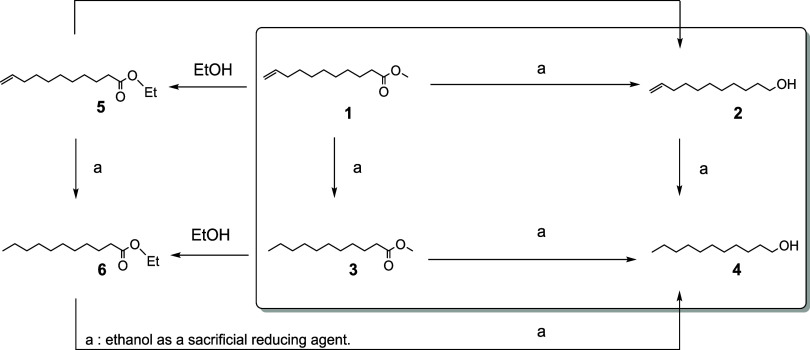
Reduction of Methyl 10-Undecenoate (**1**) into 10-Undecen-1-ol (**2**) and By-products by Transfer Hydrogenation

In a previous work,^31^ complexes **Ru-2** and **Ru-3** were tested as catalysts for ester reduction under TH conditions. It was found that using ethyl hexanoate as a model substrate and potassium tert-butoxide as a promoter in toluene, ethanol was found to be a better sacrificial hydrogen source than isopropanol or benzyl alcohol. Using substrate **1**, we first compared ruthenium complexes **Ru-1**, **Ru-2**, and **Ru-3** with sodium methoxide as a promoter in toluene employing a 20-fold excess of ethanol with respect to the substrate. From the results collected in Table 1, entries 1–3, it is evident that **Ru-1** has low efficiency and afforded only 15% of hydrogenated products (**2**, its isomers, and **4**) after 24 h at 80 °C. The main products were due to the transesterification of **1** with ethanol, accompanied by C**=**C isomerization. **Ru-2** proved to be a better catalyst for the reduction of **1** and gave a high conversion for hydrogenated products (entry 2). Again, C**=**C isomerization competed with ester reduction, eroding the selectivity for **2**. **Ru-3** was also efficient in the reduction of **1**; however, the saturated alcohol **4** was formed in about the same proportion as **2** (and its isomers) after 24 h (entry 3). Considering that toluene is regarded as a problematic solvent from the sustainability point of view, we decided to test other solvents, targeting those with good sustainability scores according to recent industrial guides.^39^ Using **Ru-2** and **Ru-3** as catalysts under otherwise the same conditions as in entries 2 or 3, the following solvents were tested: tetrahydrofuran (THF), 2-methyl-tetrahydrofuran (2-Me-THF), *p*-propylanisole (PPA), cyclopentyl methyl ether (CPME), and a 2:1 mixture of *p*-cymene/menthane (see SI Table S1). Regardless of the solvent used, the yield of the fully hydrogenated product (**4**) was always higher than that of **2** with **Ru-3** vs **Ru-2**. CPME and anisole, which are among the best solvents according to the sustainability guides, gave the best yields for **2** with **Ru-2** as the catalyst (Table 1, entries 4 and 5, respectively), and the C**=**C bond isomerization was considerably reduced in these solvents. Using isopropanol as a sacrificial hydrogen source instead of ethanol led mainly to transesterification products (entries 1 and 3, SI Table S2).

**Table 1:** Reduction of Methyl 10-Undecenoate (**1**) by Transfer Hydrogenation^a^

				yield (%)^b^				
entry	catalyst	solvent	conv^b^ (%)	**2**	C=C isomers of **2**	**4**	**5**	C=C isomers of **5**
1	**Ru-1**	toluene	100	1	11	2	5	81
2	**Ru-2**	toluene	100	4	71	16	0	9
3	**Ru-3**	toluene	100	2	46	46	0	6
4	**Ru-2**	CPME	100	51	29	10	0	10
5	**Ru-2**	anisole	100	52	29	10	0	9
6	**Os-1**	anisole	100	80	3	2	9	6
7	**Os-2**	anisole	100	74	2	5	17	2
8	**Os-3**	anisole	98	65	2	1	26	4
9^c^	**Os-3**	anisole	89	15	3	2	59	10

a Conditions: methyl 10-undecenoate (1) (0.133 mmol), catalyst = 1 mol %, NaOMe = 5 mol %, ethanol (2.66 mmol), 24 h, and 80 °C.

b Conversion and yield obtained by GC using undecane (0.064 mmol) as the internal standard.

c Without NaOMe.

The osmium catalysts **Os-1**, **Os-2**, and **Os-3** have been previously tested for the selective hydrogenation of esters, including ω-unsaturated ones.^40^ As far as we are aware, they have never been tested for ester reduction under transfer hydrogenation (TH) conditions. Table 1 (entries 6–9) shows the outcome of the reactions when the osmium catalysts were tested under the optimized conditions found for **Ru-2** (Table 1, entry 5). As observed, all osmium complexes showed a low C**=**C bond isomerization activity (only 2–3% of isomerization products were formed). While the conversions were close to 100%, the order of activity was **Os-1** > **Os-2** > **Os-3** according to the yield of **2** + **8**, with the best yield (80%) for **2** obtained with **Os-1** in entry 6. Given that **Os-3** is presumably formed from **Os-1** and a base under catalytic conditions, we decided to test the activity of **Os-3** in the absence of NaOMe in entry 9. The complex showed some activity in the reduction, but the major product was the transesterification product (**5**), demonstrating that **Os-3** itself is an active transesterification catalyst. The superior performance of osmium catalysts may offset their higher cost and scarcity, although toxicity and mining-related issues should be considered in terms of overall process sustainability.

To further explore the scope of the methodology, we decided to test the selectivity of the catalysts shown in Figure 1 for the TH to α,β-unsaturated aldehydes. We chose aldehydes (1*R*)-(−)-myrtenal (**7**) and (*E*)-cinnamaldehyde (**11**) as test substrates because the reductions of their carbonyl groups generate allylic alcohols of significant industrial interest.^41^ To the best of our knowledge, the selective reduction of myrtenal (**7**) to myrtenol (**8**) under TH conditions has not been reported. In myrtenal, both the C**=**C and C**=**O bonds can be reduced, as depicted in Scheme 2, although the former is sterically more encumbered. The results are listed in Table 2.

**Scheme 2:**
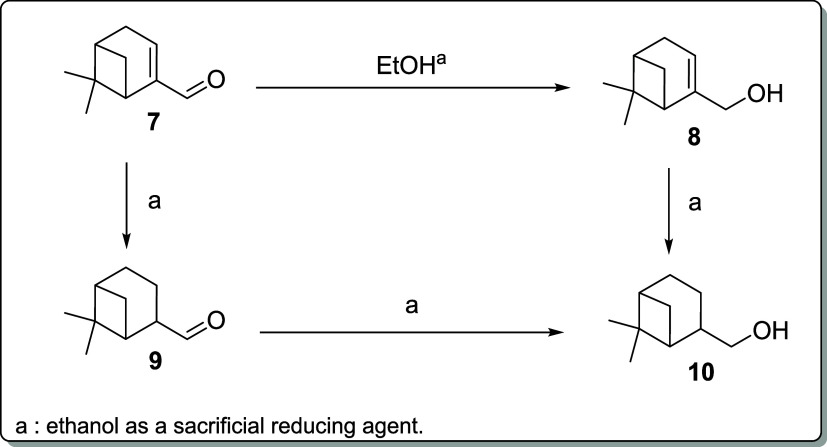
TH of Myrtenal (**7**) into Myrtenol (**8**)

**Table 2:** Reduction of Myrtenal (**7**) by Transfer Hydrogenation at a Catalyst Loading of 1 mol %^a^

			yield (%)^b^		
entry	catalyst	conv^b^ (%)	**8**	**10**	others
1	**Ru-2**	100	100	0	0
2	**Ru-3**	0	0	0	0
3	**Os-1**	100	89	11	0
4	**Os-2**	98	86	12	0
5	**Os-3**	96	85	11	0
6^c^	**Os-3**	99	99	0	0

a Reaction Conditions: Myrtenal (0.133 mmol), catalyst (1 mol %), Anisole (3 mL), EtOH (2.66 mmol), NaOMe (5 mol %), ca. 5 min, and ca. 30 °C.

b Conversion and yield obtained by GC using undecane (0.064 mmol) as the internal standard.

c Without base.

We found that some of the catalysts afforded the selective reduction of the carbonyl group in less than 5 min at ca. 30 °C. **Ru-2** delivered a quantitative yield of **8**, while **Ru-3** was not active under such mild conditions and short time. Employing NaOMe as a promoter, **Os-1**, **Os-2**, and **Os-3** delivered good yields, although **10** was formed in ca. 11% yield. It is important to mention that **Os-3** was highly active in the absence of a base (Table 2, entry 6), producing the selective formation of vinylic alcohol **8** in 99% yield.

Considering the superior activity of **Ru-2** under mild conditions, we decided to reduce the catalyst loading to 0.1 mol % and monitor the progress of TH of **7**. The time-dependent reaction profiles of the catalytic reactions carried out with **Ru-2** in different solvents are shown in Figure 2. No induction period was observed regardless of the solvent used. It is also clear that anisole is a better solvent for this reaction than CPME or toluene. Using the data from the initial linear substrate consumption, we calculated turnover frequencies (TOF) of 740 h^–1^ in anisole, 273 h^–1^ in CPME, and 160 h^–1^ in toluene. It is worth mentioning that in toluene, some C**=**C reduction to form **10** takes place in parallel with C**=**O reduction.

**Figure 2:**
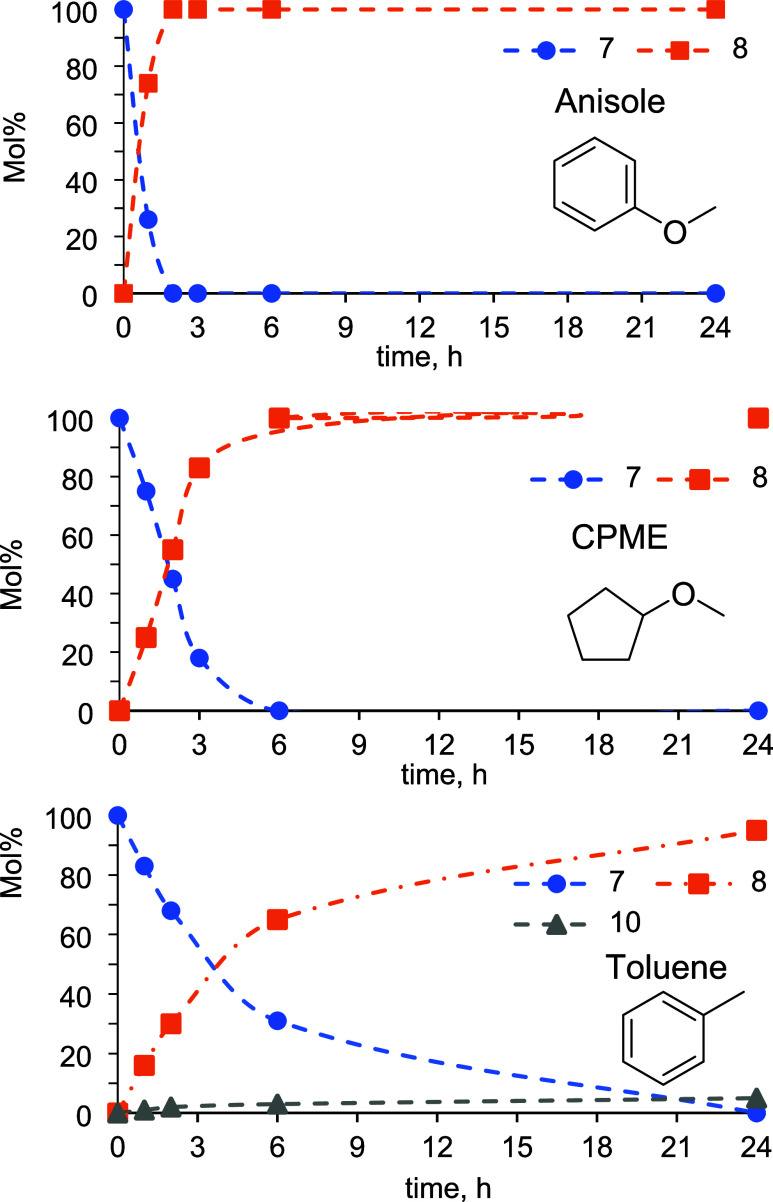
Time-dependent reaction profiles of the reduction of **7** under the TH conditions in different solvents. Conditions: Myrtenal **7** (0.665 mmol), **Ru-2** (0.1 mol %), Solvent (15 mL), EtOH (13.25 mmol), NaOMe (5 mol %), and 35 °C (dashed lines are added to aid visualization and do not represent a data fitting).

Table 3 shows the results obtained when osmium catalysts **Os-1**, **Os-2**, and **Os-3** were tested in anisole under the reaction conditions of Figure 2. From the analysis of these results, it is possible to conclude that **Os-1** is less active and selective than **Ru-2**, but **Os-2** is far more active, resulting in a turnover frequency (TOF) of at least 12,000 h^–1^ under such mild conditions. **Os-3** is also more active, resulting in a turnover rate of 5884 h^–1^. These results indicate that the catalytically active species is formed readily when the precatalysts are mixed with sodium methoxide, as observed before.^18–20^

**Table 3:** Reduction of Myrtenal (**7**) by Transfer Hydrogenation at a Catalyst Loading of 0.1 mol %^a^

				yield (mol %)^b^		
entry	catalyst	time	conv^b^ (mol %)	**8**	**10**	others
1	**Os-1**	5 min^c^	5	5	0	0
		1 h	20	16	0	4
2	**Os-2**	5 min^c^	100	100	0	0
		1 h	100	100	0	0
3	**Os-3**	5 min^c^	49	49	0	0
		1 h	90	90	0	0

a Reaction conditions: Myrtenal (0,133 mmol), catalyst (0.1 mol %), Anisole (3 mL), EtOH (2.66 mmol), NaOMe (5 mol %), 1 h, and 35 °C.

b Conversion and yield obtained by GC using undecane (0.064 mmol) as an internal standard.

c Time to quench the reaction at ca. 5 min and ca. 30 °C.

Cinnamaldehyde (**11**) has been used as a model for the selective hydrogenation of α,β-unsaturated aldehydes in a large number of studies, some reporting excellent selectivity for cinnamyl alcohol.^42–46^ We tested **Ru-2**, **Ru-3**, **Os-1**, **Os-2**, and **Os-3** (catalyst loading of 1 mol %) for transfer hydrogenation with ethanol (20 equiv) as a sacrificial hydrogen source and anisole as solvent (Table 4). In addition to the desired selective reduction to give **12**, the oxidative esterification to form **15** was also observed, along with the C**=**C reductions to form **13**, **14**, or **16** (Scheme 3). The reductions of **15** to **12** or **16** to **14** are, in principle, possible.

**Scheme 3:**
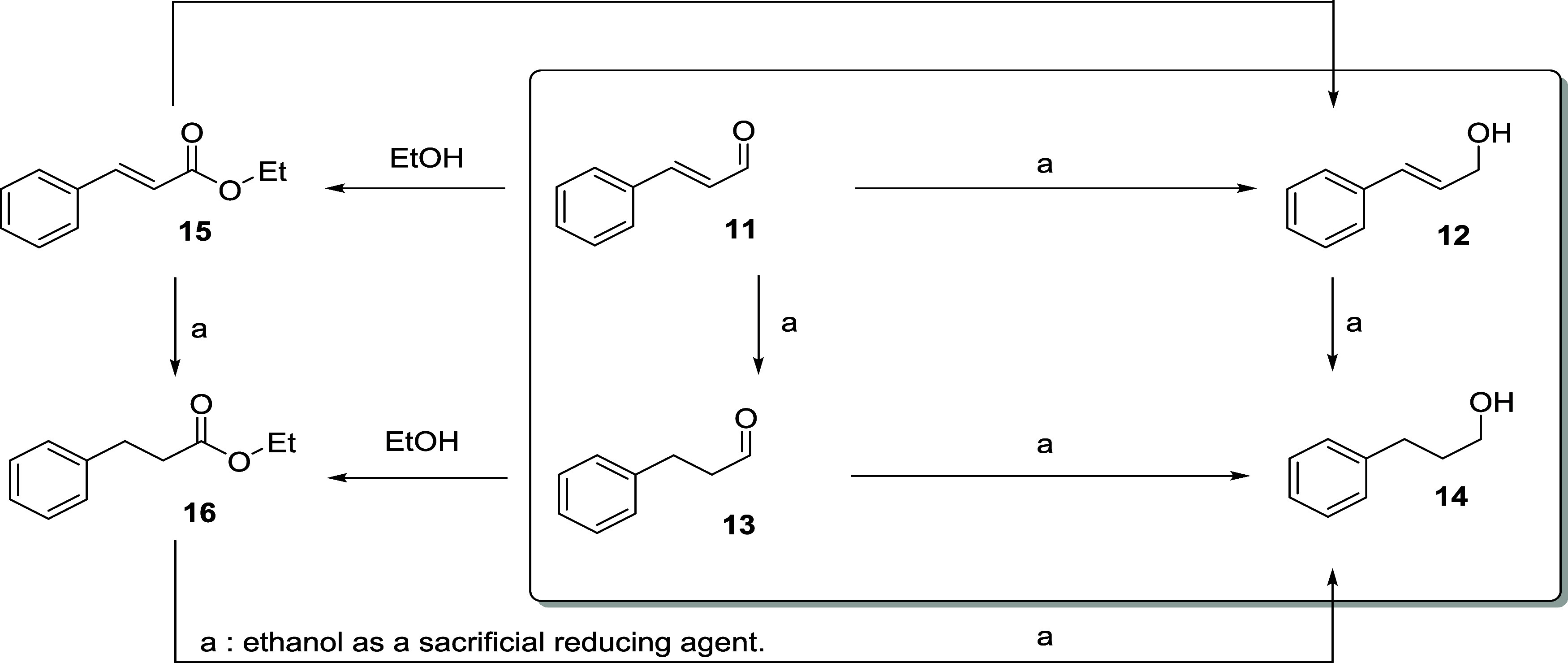
Transfer Hydrogenation of Cinnamaldehyde (**11**)

**Table 4:** Transfer Hydrogenation of Cinnamaldehyde^a^

				yield (%)^b^				
entry	catalyst	time	conv^b^ (%)	**12**	**14**	**15**	**16**	others
1	**Ru-2**	5 min^c^	99	51	0	48	0	0
		24 h	100	43	16	1	39	0
2	**Ru-3**	5 min^c^	100	57	0	43	0	0
		24 h	100	26	55	0	15	3
3	**Os-1**	5 min^c^	100	89	4	0	8	0
		24 h	100	0	90	0	4	6
4	**Os-2**	5 min^c^	100	75	12	0	13	0
		24 h	100	0	86	0	8	6
5	**Os-3**	5 min^c^	100	8	80	0	12	0
		24 h	100	0	89	0	5	6
6^d^	**Os-3**	5 min^c^	100	98	2	0	0	0
		24 h	100	8	77	0	11	4

a Reaction conditions: **11** (0.133 mmol), catalyst (1 mol %), Anisole (3 mL), EtOH (2.65 mmol), NaOMe (5 mol %), and 80 °C.

b Conversion and yield obtained by GC using undecane (0.064 mmol) as the internal standard.

c Time to quench the reaction at ca. 5 min and ca. 30 °C.

d Without base.

When the TH was carried out at 30 °C, a full conversion of **11** took place after 5 min for all of the catalysts used (Table 4). With **Ru-2** or **Ru-3** (entries 1 and 2) as catalysts, **15** was initially observed. When the reaction was performed at 80 °C and the reaction time increased to 24 h, then **12** was partly reduced to **14** and **15** to **16**. This behavior suggests a kinetic pathway in which the oxidative esterification of compound **11** is the source of hydrogen. With the osmium catalyst **Os-1**, **Os-2**, or **Os-3** (entries 3–5), **15** was not observed, and the main reduction product after 5 min was the desired **12**. At 80 °C and after 24 h, further reduction of **12** to form **14** and a small amount of **16** took place. Noteworthy is that **Os-3** promotes the selective reduction of **11** to **12** in only 5 min, in the absence of NaOMe (entry 6).

Given the high activity of the osmium catalysts, we decided to test them at a lower catalyst loading (0.1 mol %) at 35 °C. From the results in Table 5, the order of the activity of the catalysts is **Os-2** > **Os-3** > **Os-1**, the same as was found for the reduction of myrtenal (Table 3). The turnover frequencies (TOFs) after 5 min of reaction were 4320, 10,920, and 5640 h^–1^ for **Os-1**, **Os-2**, and **Os-3**, respectively. Under these reaction conditions, **Os-3** was not active in the absence of NaOMe (entry 4). All osmium catalysts produced small amounts of **15**, thus resulting in a small selectivity loss.

**Table 5:** Transfer Hydrogenation of **11** at a Catalyst Loading of 0.1 mol %^a^

				yield (%)^b^				
entry	catalyst	time	conv^b^ (%)	**12**	**14**	**15**	**16**	others
1	**Os-1**	5 min^c^	36	29	0	3	0	4
		1 h	100	91	0	4	0	5
2	**Os-2**	5 min^c^	91	81	0	10	0	0
		1 h	100	89	0	11	0	0
3	**Os-3**	5 min^c^	47	38	0	5	0	4
		1 h	100	85	0	9	0	6
4^d^	**Os-3**	5 min^c^	0	0	0	0	0	0
		1 h	1	1	0	0	0	0

a Reaction conditions: cinnamaldehyde **11** (0,133 mmol), catalyst (0.1 mol %), Anisole (3 mL), EtOH (2.65 mmol), NaOMe (5 mol %), and 35 °C.

b Conversion and yield obtained by GC using undecane (0.064 mmol) as the internal standard.

c Time to quench the reaction at ca. 5 min and ca. 30 °C.

d Without base.

To assess the activity of the osmium catalysts at lower catalyst loadings, we tested **Os-1**, **Os-2**, and **Os-3** at 0.02 mol % (200 ppm). The results are presented in Section S5. These catalysts are rather sensitive to water, and trace amounts of water on solvents or substrates drastically reduce the conversion and selectivity of the catalysts at low catalyst loadings, as observed in Tables S4 and S5 (SI).

To assess the efficiency of the catalysts in the reduction of conjugated esters, we tested them using trans-cinnamic acid methyl ester as a model substrate. The results are presented in the Supporting Information, Section S4. The system promoted rapid esterification with ethanol, even at 30 °C; however, none of the catalysts selectively reduced the carboxylic group. **Ru-3**, **Os-1**, and especially **Os-2** facilitated the reduction of the C**=**C double bond. Only after the conjugation was disrupted were the catalysts able to reduce the carboxylic group, ultimately yielding 3-phenylpropanol (88%) after 24 h at 80 °C.

## Conclusions

This work reports a greener alternative to the conventional hydrogenation of unsaturated esters and α,β-unsaturated aldehydes by using green solvents, a sustainable sacrificial hydrogen source employing pincer-type **Ru** and **Os** catalysts, the latter tested for the first time for this kind of reaction. For methyl undecenoate, the osmium catalysts were able to selectively reduce the C=O bond while retaining the C=C bond and avoiding undesired isomerization. For α,β-unsaturated aldehydes, the selection of catalysts in combination with reaction conditions allowed for the selective reduction of the C=O bond in excellent yields at room temperature, at reasonably low catalyst loadings. We hope that this contribution may encourage further research toward greener and more sustainable catalytic processes for carbonyl reduction.

## Experimental Section

### General Methods

All experiments and manipulations of air- or water-sensitive compounds were carried out under an argon atmosphere using a glovebox (MBRAUN UNILAB PRO) or using the standard Schlenk line techniques. All reactants and solvents were purchased from Sigma-Aldrich and used as received, unless otherwise stated (see Section S1 in SI for details). **Ru-1** was purchased from Sigma-Aldrich (95%) and used as received. **Ru-2** and **Ru-3** (also available from Sigma-Aldrich) were synthesized according to reported procedures. **Os-1**, **Os-2**, and **Os-3** were synthesized according to reported procedures.^47^ The products were characterized by gas chromatography coupled to mass spectrometry (GC-MS) and ^1^H and ^13^C nuclear magnetic resonance (NMR), as well as co-injection of authentic samples with the products on gas chromatography (see Section S7 in SI). Routine quantitative product analyses were made by gas chromatography (GC) employing undecane as an internal standard (see Section S1 in SI for details).

### General Procedures for Transfer Hydrogenation

A 4 mL vial was filled inside the glovebox with the corresponding substrate (0.133 mmol), catalyst (0.0266–1.33 × 10^–3^ mmol), sodium methoxide (6.65 × 10^–3^ mmol), ethanol (0.15 mL), solvent (3.0 mL), undecane (0.064 mmol), and a magnetic stirring bar. The vial was closed with a cap containing a septum, removed from the glovebox, and fitted on an aluminum block over a Corning 6795–420D hot plate/stirring apparatus with an external sensor inside the aluminum block to control the temperature. The reaction was quenched by opening the vial in the air, adding trifluoroacetic acid (6.65 × 10^–3^ mmol) to neutralize the alkoxides, and diluting with untreated toluene for GC analyses.

## Supplementary Material



## Notes

Methodology and analytical details; green solvent screening; hydrogen source screening; catalyst loading reduction study; TH of cinnamic acid; methyl ester; NMR and MS of the products; and representative GC trace analysis (PDF)
