# Supplementary material for: Selective Carbonyl Reduction in Unsaturated Esters and Aldehydes by Transfer Hydrogenation
Source: Organometallics. 2025 Jul 17;44(14):1538–44. doi: 10.1021/acs.organomet.5c00094 (PMC12309145; doi:10.1021/acs.organomet.5c00094)
Supplement: Supplementary file 1 [file om5c00094_si_001.pdf]

# Electronic Supporting Information

## Selective Carbonyl Reduction in Unsaturated Esters and Aldehydes by Transfer Hydrogenation

Víctor Martínez-Agramunt,[‡,†] Lucas H. R. Passos,[†] Dmitry G. Gusev,[§] Eduardo Peris[‡] and Eduardo N. dos Santos*[,†]

[†]Departamento de Química. Universidade Federal de Minas Gerais (UFMG). Av. Antônio Carlos 6627, MG 31270-901, Belo Horizonte (Brazil).

[‡]Institute of Advanced Materials (INAM) Universitat Jaume I (UJI). Av. Vicente Sos Baynat s/n, CV 12071, Castellón (Spain).

[§]Wilfrid Laurier University (WLU) Waterloo, Ontario N2L 3C5 (Canada).

*corresponding author
e-mail: nicolau@ufmg.br

**S1 General Comments** **S3**

**S2 Solvent Screening** **S4**

**S3 Sacrificial Hydrogen Source Screening** **S5**

**S4 Transfer Hydrogenation of methyl cinnamate (18)** **S6**

**S5 Reactions Performed at Catalyst Loading of 0.02 mol%** **S7**

**S6 NMR & GC-MS Identification of Products** **S8**

**S7 References** **S20**

## S1 General Comments

All experiments and manipulations of air or water- sensitive compounds were carried out under argon atmosphere using a glove box (MBRAUN UNILAB PRO) or using the standard Schlenk line techniques. The identification of some substances such as 10-methyl undecane and undecen-1-ol were made by co-injection with authentic samples. **Gas Chromatography (GC) analyses** were performed on samples diluted with untreated toluene on Shimadzu GC2010 Plus instrument equipped with an auto-sampler, fitted with polar Carbowax column (30 m length, 0.25 mm internal diameter, 0.25 µm film thickness) and a flame ionization detector (FID). Conversion and selectivity were determined using undecane as internal standard. Carrier gas: $H_2$ (UHP grade); Makeup gas: $N_2$ (UHP grade). CG and column conditions: injector temperature 230°C, split ratio 1:100; detector temperature 240°C; oven temperature, 40 °C; hold time, 5 min, 17 °C/min to 240°C, held for 7 minutes. Pressure of $H_2$ of 88.1 KPa with total flow of 254.8 mL/min, column flow 2.5 $mL.min^{-1}$ and linear speed of 60.6 cm/s. Injected volume 1.0 µL. Integration by CGSolution software. THF (Tetrahydrofuran) (≥99.9%, anhydrous, Sigma-Aldrich), anisole (≥99.7%, anhydrous, Sigma-Aldrich) and toluene (≥99.8%, anhydrous, Sigma-Aldrich) were purchased from Sigma Aldrich and were opened inside a glove box. Methyl 10-undecenoate (96%, Sigma-Aldrich), (E)-cinnamaldehyde (99%, Sigma-Aldrich), (1R)-(-)-myrtenal (98%, Sigma-Aldrich), undecane (≥99, Sigma-Aldrich), were treated with Magnesol® (5 % m/m) and alumina (5 % m/m), and heated to 80 °C under vigorous stirring by 2 h, then distilled in a Kugelrohr distillation apparatus at reduced pressure, collected under argon atmosphere and store in a glovebox prior to use. **Ru-1** was purchased from Sigma Aldrich (95%) and used as received. **Ru-2**[1], **Ru-3**[2], (also available in Sigma Aldrich) were synthesized according to reported procedures. **Os-1**, **Os-2** and **Os-3** were synthesized according to reported procedures.[3]

## S2 Solvent Screening

In this work, a comparative study of up to 10 different solvents was done. Among them, Solketal, Dihidroeugenol, Cyrene, 1,3-Dimethyl-2-imidazolidinone (DMI), were proven to not be suitable for the Transfer Hydrogenation reaction, since very low conversions (below 5% were observed). Besides the ones commented in the manuscript (Anisole, CPME and Toluene), THF, 2-Methyl THF, p-propyl Anisole (PPA) and a mixture of p-Cymene/p-Mentane (pCy/pMe), gave the results shown in Table S1.

**Table S1** Comparative study with **Ru-2** and **Ru-3** for the Transfer Hydrogenation of **1** in selected solvents.[a]

| Entry | Cat. | Solvent | Conv[b] | Yield (%)[b] | | | | |
|---|---|---|---|---|---|---|---|---|
| | | | | **2** | Isomerized unsaturated alcohols | **4** | **5** | Isomerized esters |
| **1** | **Ru-2** | Anisole | 100 | 52 | 31 | 10 | 0 | 6 |
| **2** | **Ru-3** | Anisole | 91 | 2 | 49 | 30 | 3 | 7 |
| **3** | **Ru-2** | CPME | 100 | 51 | 33 | 10 | 0 | 6 |
| **4** | **Ru-3** | CPME | 100 | 6 | 65 | 21 | 0 | 8 |
| **5** | **Ru-2** | Toluene | 100 | 4 | 72 | 15 | 0 | 7 |
| **6** | **Ru-3** | Toluene | 100 | 2 | 44 | 44 | 0 | 9 |
| **7** | **Ru-2** | THF | 100 | 2 | 51 | 9 | 1 | 37 |
| **8** | **Ru-3** | THF | 100 | 1 | 37 | 35 | 1 | 26 |
| **9** | **Ru-2** | 2Me-THF | 100 | 2 | 75 | 4 | 1 | 18 |
| **10** | **Ru-3** | 2Me-THF | 100 | 2 | 54 | 35 | 1 | 9 |
| **11** | **Ru-2** | PPA | 100 | 4 | 74 | 13 | 0 | 9 |
| **12** | **Ru-3** | PPA | 100 | 2 | 51 | 36 | 0 | 11 |
| **13** | **Ru-2** | *p*Cym/*p*Men | 100 | 4 | 62 | 20 | 0 | 9 |
| **14** | **Ru-3** | *p*Cym/*p*Men | 100 | 7 | 54 | 24 | 0 | 15 |

[a]Conditions: methyl 10-undecenoate (**1**) (0.133 mmol), **Ru-cat** (1 mol%), Solvent (3 mL), EtOH (2.66 mmol), NaOMe (5 mol%); 24 h; 80 °C. [b]Conversion and yield obtained by GC using undecane (0.064 mmol) as internal standard. CPME = Cyclopentyl methyl ether. THF = Tetrahidrofuran. 2Me-THF = 2-Methyltetrahidrofuran. PPA= *p*-propyl Anisole. *p*Cym = *p*-Cymene. *p*Men = *p*-Menthane.

## S3 Sacrificial Hydrogen Source Screening

In this work, once Anisole was proven to be the most suitable solvent, due to the highest yield when used, combined with the highest sustainability rank according to GSK Solvent Guide; the hydrogen source was tested. For this purpose, we tested both ethanol and isopropanol as sacrificial hydrogen source as displayed in table S2.

**Table S2** Comparative test with **Ru-2** for the Transfer Hydrogenation of **1** in selected solvents.[a]

| Entry | Hydrogen Source | Solvent | Conv[b] | Yield (%)[b] | | | | |
|---|---|---|---|---|---|---|---|---|
| | | | | **2** | Isomerized unsaturated alcohols | **4** | **5** | Isomerized esters |
| **1** | EtOH (2.66 mmol) | Anisole (3mL) | 100 | 53 | 31 | 10 | 0 | 6 |
| **2** | EtOH (17.73 mmol) | Anisole (2mL) | 100 | 37 | 27 | 18 | 0 | 18 |
| **3** | iPrOH (2.66 mmol) | Anisole (3mL) | 100 | 0 | 13 | 0 | 0 | 83 |
| **4** | iPrOH (17.73 mmol) | Anisole (2mL) | 100 | 0 | 7 | 1 | 0 | 89 |

[a]Conditions: methyl 10-undecenoate (**1**) (0.133 mmol), **Ru-2** (1 mol%), NaOMe (5 mol%); 24 h; 80 °C.
[b]Conversion and yield obtained by GC using undecane (0.064 mmol) as internal standard

## S4 Transfer Hydrogenation of methyl cinnamate (18)

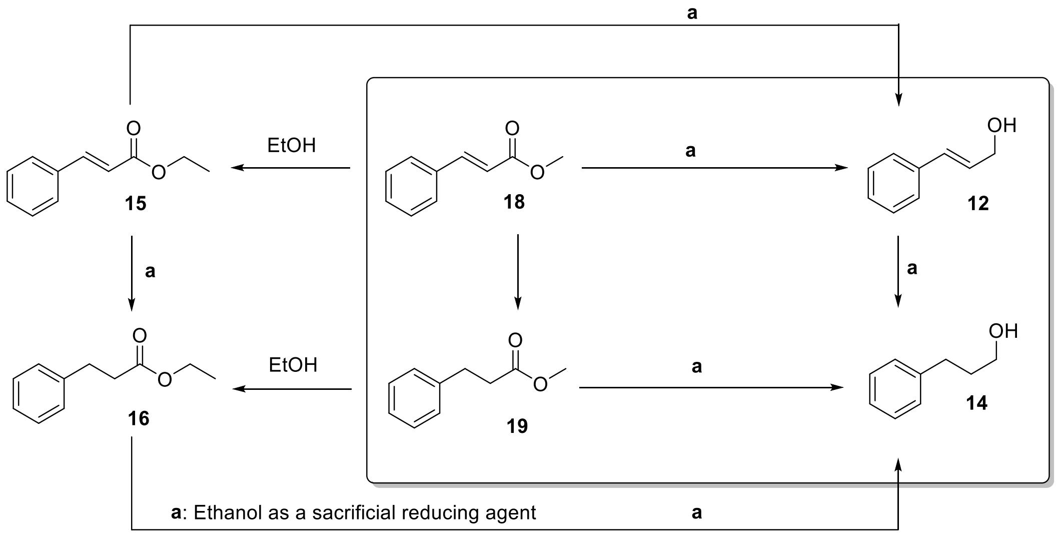


**Table S3** Reduction of methyl cinnamate (**18)** under Transfer Hydrogenation with selected catalysts.[a]

| Entry | Catalyst | Time | Conv.[b] (%) | Yield (%)[b] | | | | | |
|---|---|---|---|---|---|---|---|---|---|
| | | | | **12** | **14** | **15** | **16** | **19** | Others |
| 1 | **Ru-2** | 5min[b] | 94 | 0 | 0 | 88 | 6 | 0 | 0 |
| | | 1h | 100 | 23 | 2 | 4 | 65 | 0 | 6 |
| | | 24h | 100 | 1 | 16 | 7 | 70 | 6 | 0 |
| 2 | **Ru-3** | 5min[b] | 93 | 0 | 0 | 91 | 0 | 0 | 2 |
| | | 1h | 99 | 24 | 37 | 5 | 29 | 2 | 3 |
| | | 24h | 99 | 11 | 69 | 1 | 9 | 0 | 9 |
| 3 | **Os-1** | 5min[b] | 94 | 0 | 0 | 74 | 18 | 1 | 1 |
| | | 1h | 97 | 0 | 0 | 33 | 58 | 6 | 0 |
| | | 24h | 100 | 0 | 83 | 0 | 7 | 1 | 9 |
| 4 | **Os-2** | 5min[b] | 94 | 0 | 0 | 72 | 20 | 1 | 1 |
| | | 1h | 97 | 0 | 0 | 30 | 61 | 6 | 0 |
| | | 24h | 100 | 0 | 88 | 0 | 5 | 0 | 7 |
| 5 | **Os-3** | 5min[b] | 93 | 0 | 0 | 77 | 13 | 1 | 2 |
| | | 1h | 97 | 0 | 0 | 41 | 51 | 4 | 1 |
| | | 24h | 98 | 0 | 0 | 26 | 62 | 5 | 5 |

[a]Reaction Conditions: Methyl Cinnamate (0.133 mmol), Catalyst (1 mol%), Anisole (3 mL), EtOH (2.66 mmol), NaOMe (5 mol%); 80 °C. [b]Conversion and yield obtained by GC using undecane (0.064) as internal standard. [b] time to quench the reaction at ca. 30ºC.

## S5 Reactions Performed at Catalyst Loading of 0.02 mol%

**Table S4** Reduction of myrtenal (**7**) by Transfer Hydrogenation at a catalyst loading of 0.02 mol%[a]

| Entry | Catalyst | Conv.[b] (%) | Yield (%)[b] | | |
|---|---|---|---|---|---|
| | | | **8** | **10** | Others |
| 1 | **Os-1** | 6 | 3 | 0.4 | 2.6 |
| 2 | **Os-2** | 14 | 10 | 1 | 4 |
| 3 | **Os-3** | 8 | 4 | 0.4 | 3.6 |

[a]Reaction Conditions: Myrtenal (0.133 mmol), catalyst (0.02 mol%), Anisole (3 mL), EtOH (2.66 mmol), NaOMe (5 mol%), 2 h, 35 °C. [b]Conversion and yield obtained by GC using undecane (0.064 mmol) as internal standard.

**Table S5** Transfer Hydrogenation of **11** at a catalyst loading of 0.02 mol%.[a]

| Entry | Catalyst | Conv.[b] (%) | Yield (%)[b] | | | | |
|---|---|---|---|---|---|---|---|
| | | | **12** | **14** | **15** | **16** | Others |
| 1 | **Os-1** | 24 | 9 | 0 | 0 | 0 | 16 |
| 2 | **Os-2** | 49 | 26 | 0 | 3 | 0 | 20 |
| 3 | **Os-3** | 37 | 16 | 0 | 0 | 0 | 21 |

[a]Reaction Conditions: Cinnamaldehyde **11** (0.133 mmol), catalyst (0.02 mol%); Anisole (3 mL), EtOH (2.66 mmol), NaOMe (5 mol%), 2h, 35 °C. [b]Conversion and yield obtained by GC using undecane (0.064 mmol) as internal standard.

## S6 NMR & GC-MS Characterization of Products

**S6.1** Product characterization for the transfer hydrogenation of methyl 10-undecenoate (**1**):

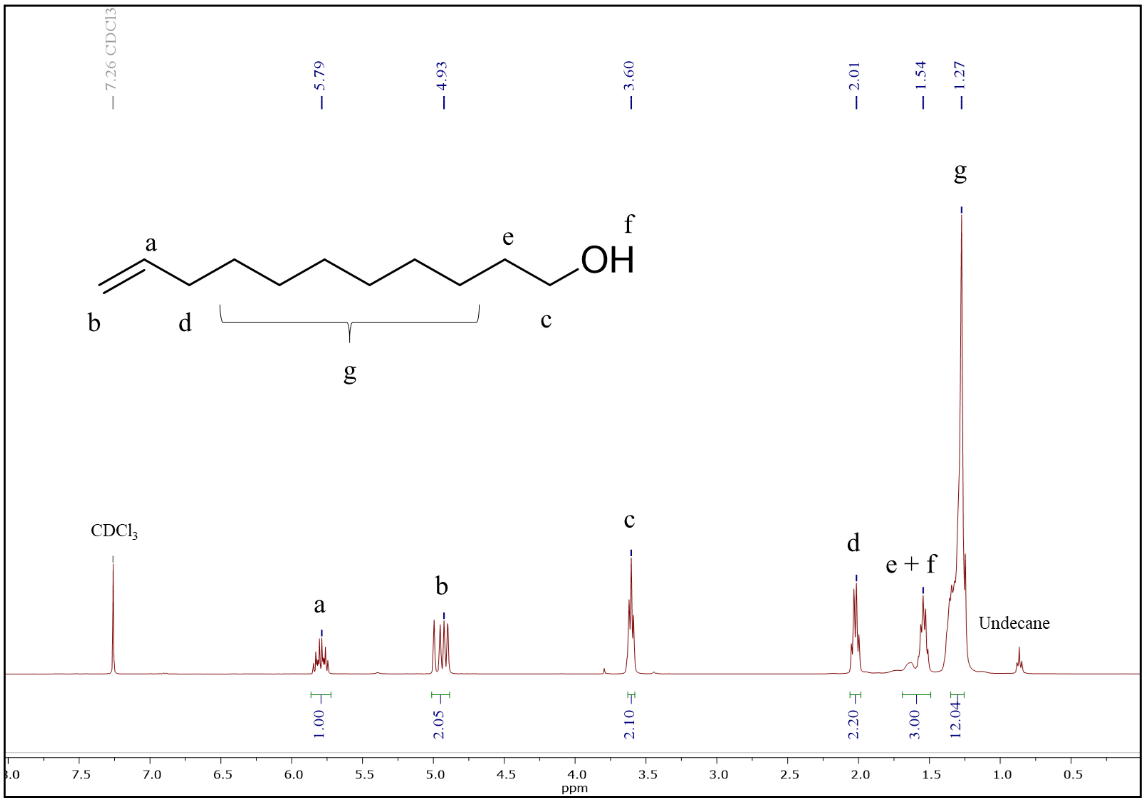


**Figure S1** $^{1}$H NMR of **2** (400 MHz, $CDCl_3$) δ = 5.79 (m, 1H, C*H*), 4.93 (dd, 2H, C$H_2$), 3.60 (t, 2H, C$H_2$), 2.01 (q, 2H, C$H_2$), 1.54 (s, 1H, O*H*) + (t, 2H, C$H_2$), 1.27 (m, 12H, C$H_2$).

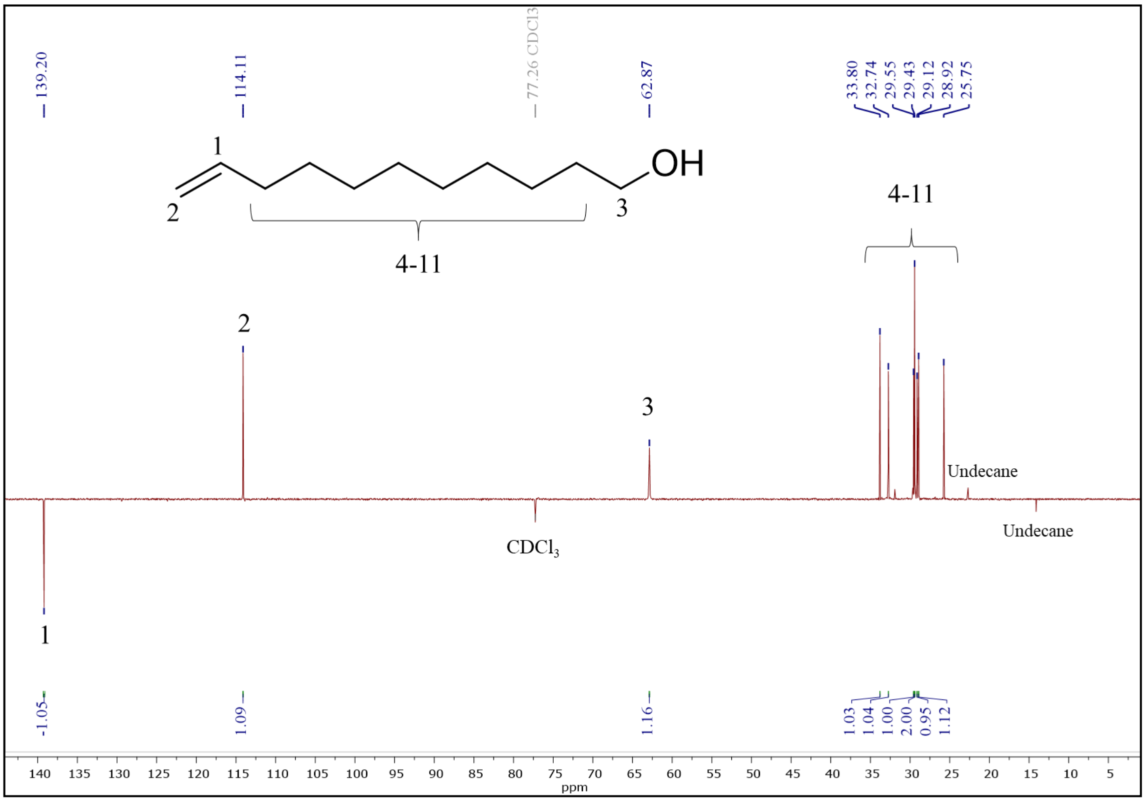


**Figure S2** $^{13}$C NMR of **2** (101 MHz, $CDCl_3$) δ 139.20 (*C*H), 114.11(*C*$H_2$), 62.87(OH-*C*$H_2$), 33.80(*C*$H_2$), 32.74(*C*$H_2$), 29.55(*C*$H_2$), 29.43(*C*$H_2$), 29.12(*C*$H_2$), 28.92(*C*$H_2$), 25.75(*C*$H_2$).

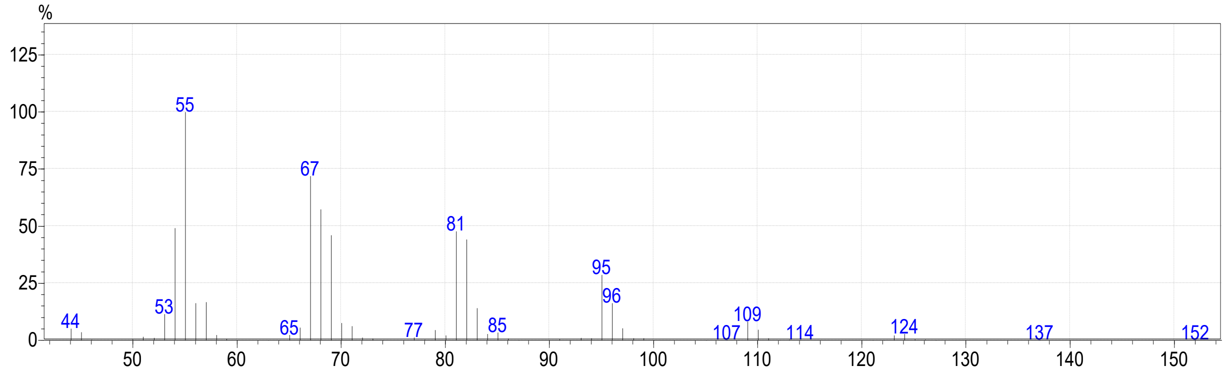


**Figure S3** Mass spectrum of **2.** Calculated for $C_{11}H_{22}O$: 170. MS (70 eV, EI): observed m/z (%): 152 (0.05) ($M^{+\cdot}$-18), 124 (3), 109 (8), 96 (16), 95 (27), 85 (3), 82 (43), 81 (47), 72 (1), 69 (46), 68 (57), 67 (72), 65 (2), 57 (16), 56 (17), 55 (100), 54 (49), 53 (11), 44 (5).

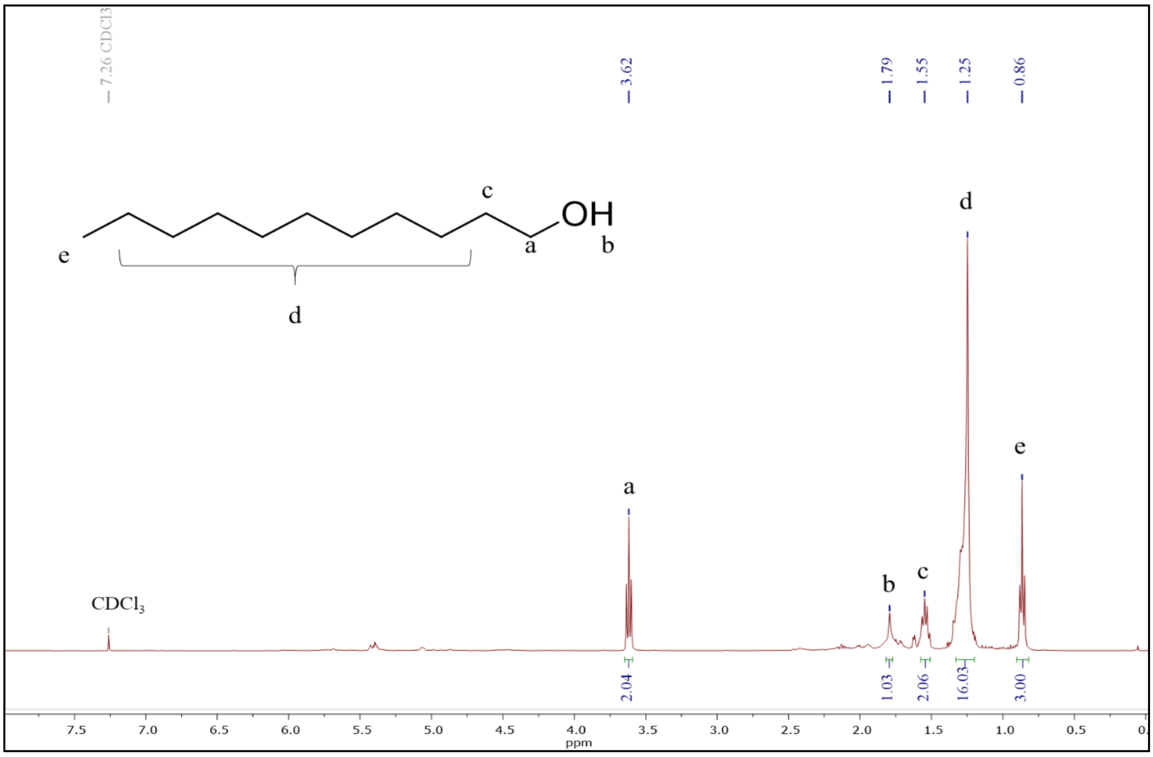


**Figure S4** $^{1}$H NMR of **4** (400 MHz, CDCl$_3$) δ 3.62 (t, 2H, C$H_2$), 1.79 (s, 1H, O*H*), 1.55 (m, 2H, C$H_2$), 1.25 (m, 16H, C$H_2$) 0.86 (t, 3H, C$H_3$).

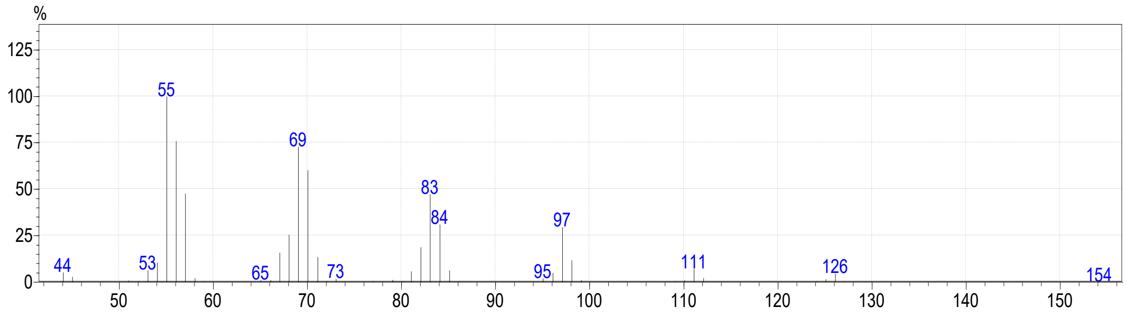


**Figure S5** Mass spectrum of **4**. Calculated for $C_{11}H_{24}O$: 172; MS (70 eV, EI) observed m/z (%): 154 (0.16) ($M^{+\cdot}$-18), 126 (5), 125 (2), 112 (2), 111 (7), 110 (1), 109 (1), 99 (1), 98 (12), 97 (30), 96 (5), 95 (2), 85 (6), 84 (31), 83 (47), 82 (19), 81 (6), 79 (1), 77 (1), 73 (2), 72 (1), 71 (14), 69 (73), 68 (26), 67 (16), 66 (1), 65 (1), 58 (2), 57 (48), 56 (76), 55 (100), 54 (11), 53 (6), 52 (1), 51 (1), 45 (3), 44 (5)

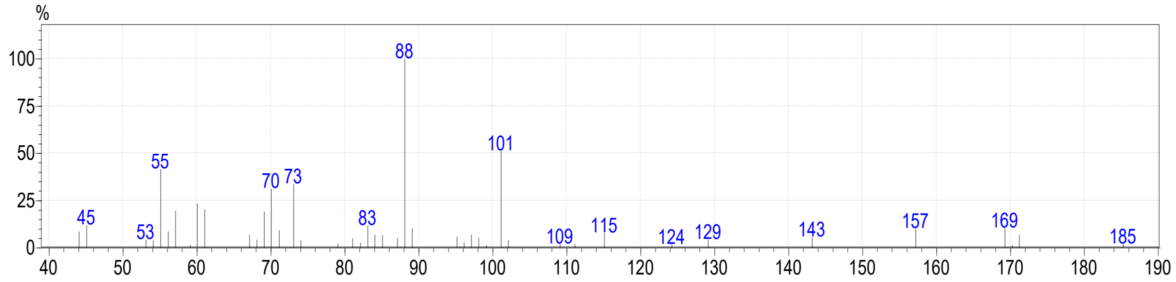


**Figure S6** Mass spectrum of C-C double-bond isomer of **5**. (rt. 10.76 min.). Calculated for $C_{13}H_{24}O_2$: 212. MS (70 eV, EI) observed m/z (%):185 (2), 171 (7), 170 (2), 169 (11), 157 (10), 143 (6), 129 (4), 115 (8), 111 (2), 109 (2), 102 (4), 101 (51), 99 (2), 98 (5), 97 (7) 96 (3), 95 (6), 89 (10), 88 (100), 87 (5), 85 (7), 84 (7), 83 (12), 82 (3), 81 (5), 79 (2), 74 (4), 73 (34), 71 (9), 70 (32), 69 (19), 68 (4), 67 (7), 61 (21), 60 (24), 59 (2), 57 (20), 56 (9), 55 (42), 54 (4), 53 (4), 45 (12), 44 (9).

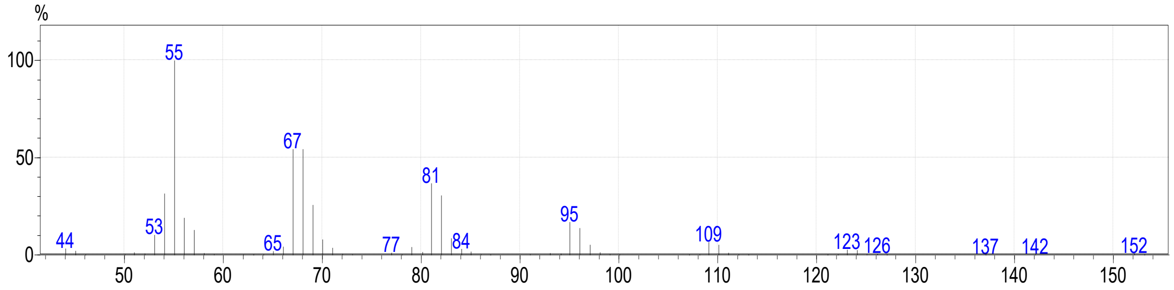


**Figure S7** Mass spectrum of C-C of Double-bond isomer of **2**. (rt. 11.52 min) Calculated for $C_{11}H_{22}O$ = 170; MS (70 eV, EI) observed m/z (%): 152 (1) ($M^{+\cdot}$-18), 142 (0.03), 137 (0.25), 123 (3), 109 (7), 96 (15), 95 (18), 85 (2), 82 (34), 81 (40), 77 (2), 69 (25), 68 (59), 67 (60), 65 (2), 56 (16), 55 (100), 54 (33), 53 (12), 44 (4)

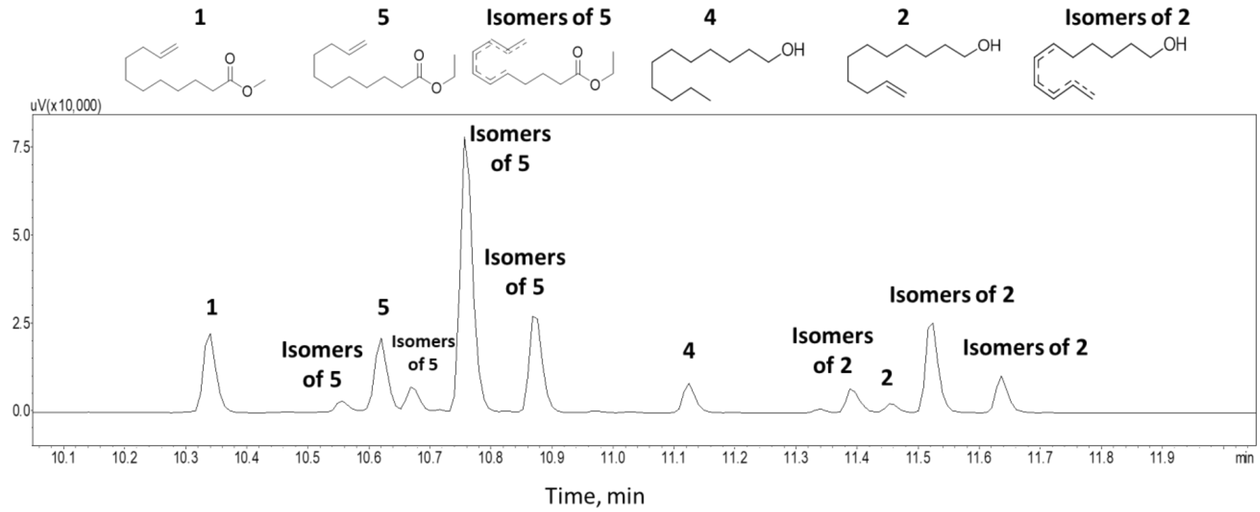


**Figure S8** Representative GC trace analysis of the transfer hydrogenation products of methyl 10-undecenoate (**1**) under GC-MS conditions of analysis

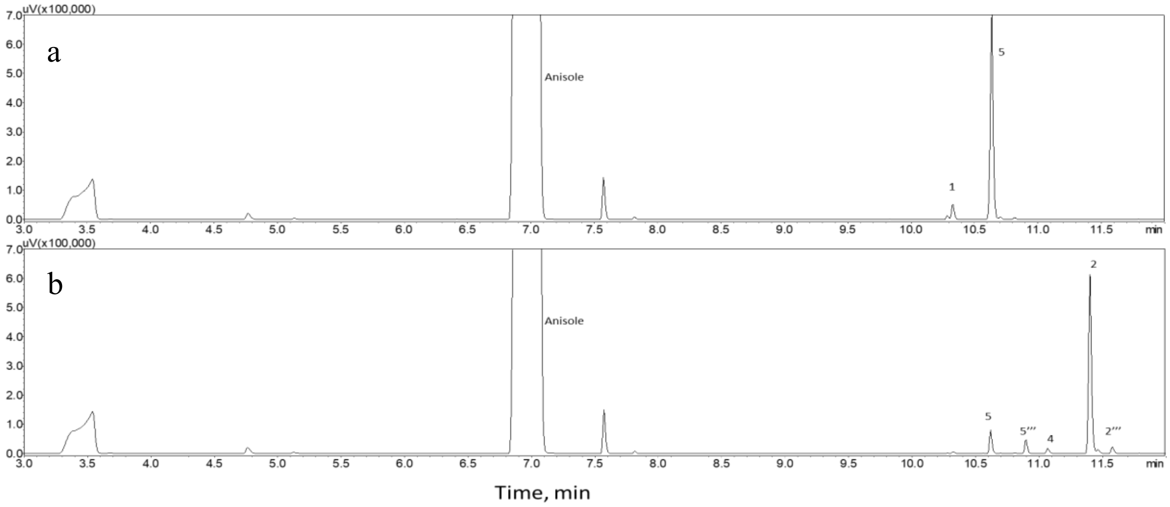


**Figure S9** Representative GC trace analysis of the transfer hydrogenation products of methyl 10-undecenoate (**1**) under GC-FID conditions described in **S1**. Entry 6 of Table 1. Conditions: methyl 10-undecenoate (1) (0.133 mmol), Anisole (3 mL), **Os-1** = 1 mol%, NaOMe = 5 mol%; ethanol (2.66 mmol), 24 h; 80 °C. a) time = 0 min (time to quench the reaction); b) time = 24h.

**Table S6** Areas of GC Analysis of the runs in Table 1 [a]

| Entry (time, h) | Internal standard | Peak (RT) | Peak (RT) | Isomers of 2 (RT) | | | | Peak (RT) | Peak (RT) | Isomers of 5 (RT) | | |
|---|---|---|---|---|---|---|---|---|---|---|---|---|
| | Undecane (3.41) | **1** (10.37) | **2** (11.44) | **2’** (11.33) | **2’’** (11.38) | **2’’’** (11.51) | **2’’’’** (11.62) | **4** (11.11) | **5** (10.66) | **5’** (10.61) | **5’’** (10.75) | **5’’’** (10.86) |
| 1 (0) | 369613 | 20340 | 0 | 0 | 0 | 0 | 0 | 0 | 308786 | 0 | 0 | 0 |
| 1 (24) | 364156 | 0 | 1810 | 0 | 0 | 19445 | 9416 | 5992 | 12574 | 7634 | 152002 | 54791 |
| 2 (0) | 22773 | 2892 | 0 | 0 | 0 | 0 | 0 | 0 | 43712 | 0 | 0 | 0 |
| 2 (24) | 161881 | 0 | 12741 | 5130 | 27857 | 139482 | 39549 | 46262 | 0 | 5530 | 14803 | 5506 |
| 3 (0) | 122896 | 10234 | 0 | 0 | 0 | 0 | 0 | 0 | 96034 | 0 | 0 | 0 |
| 3 (24) | 354069 | 0 | 4266 | 0 | 4488 | 70176 | 44559 | 119475 | 0 | 8887 | 4365 | 3178 |
| 4 (0) | 161535 | 27048 | 0 | 0 | 0 | 0 | 0 | 0 | 326614 | 0 | 0 | 0 |
| 4 (24) | 163138 | 0 | 135582 | 32039 | 12788 | 34873 | 0 | 26862 | 0 | 4497 | 7859 | 13178 |
| 5 (0) | 160899 | 14583 | 0 | 0 | 0 | 0 | 0 | 0 | 302097 | 0 | 0 | 0 |
| 5 (24) | 214238 | 0 | 176850 | 15645 | 12103 | 68637 | 0 | 34558 | 0 | 0 | 13993 | 15331 |
| 6 (0) | 1323463 | 99578 | 0 | 0 | 0 | 0 | 0 | 0 | 1112652 | 0 | 0 | 0 |
| 6 (24) | 1366744 | 0 | 943126 | 0 | 0 | 32263 | 0 | 27995 | 110221 | 0 | 0 | 70101 |
| 7 (0) | 1320687 | 71085 | 0 | 0 | 0 | 0 | 0 | 0 | 1176114 | 0 | 0 | 0 |
| 7 (24) | 1301892 | 0 | 856721 | 0 | 0 | 0 | 27097 | 23975 | 199416 | 0 | 0 | 57051 |
| 8 (0) | 1104860 | 96061 | 0 | 0 | 0 | 0 | 0 | 0 | 932195 | 0 | 0 | 0 |
| 8 (24) | 1192621 | 28839 | 790948 | 0 | 0 | 25840 | 0 | 16427 | 312981 | 0 | 0 | 47101 |
| 9[b] (0) | 1372667 | 448374 | 0 | 0 | 0 | 0 | 0 | 0 | 1034497 | 0 | 0 | 0 |
| 9[b] (24) | 1339236 | 146345 | 195422 | 0 | 0 | 15710 | 16177 | 26472 | 780628 | 0 | 68867 | 64208 |

[a]Conditions: methyl 10-undecenoate (1) (0.133 mmol), Catalyst = 1 mol %, NaOMe = 5 mol%; ethanol (2.66 mmol), 24 h; 80 °C. [b]Without NaOMe. RT= retention time

**S6.2** Product characterization for the transfer hydrogenation of myrtenal (**7**)

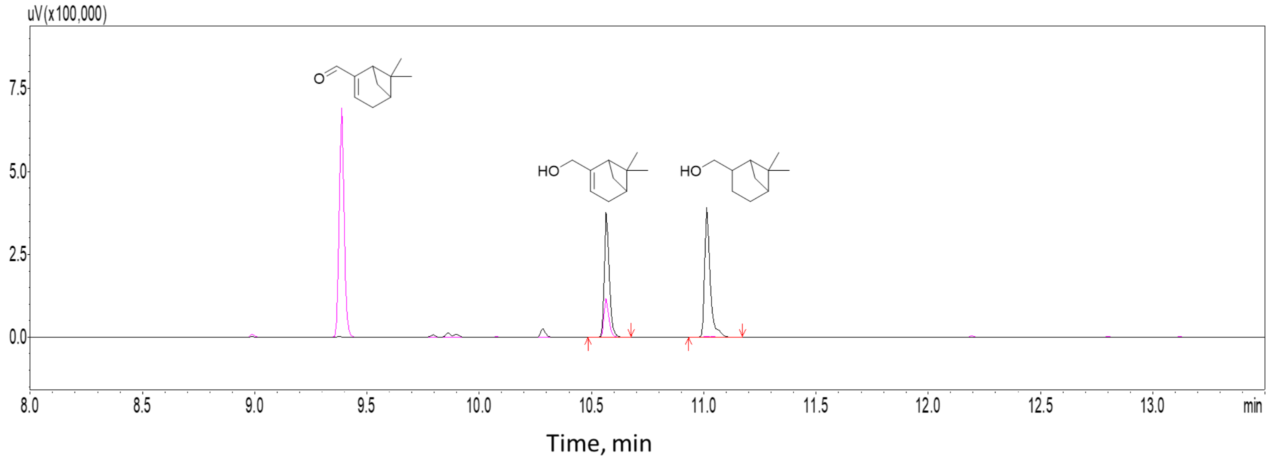


**Figure S10** Representative GC trace analysis for the transfer hydrogenation products of myrtenal (**7**), characterized by GC-MS .

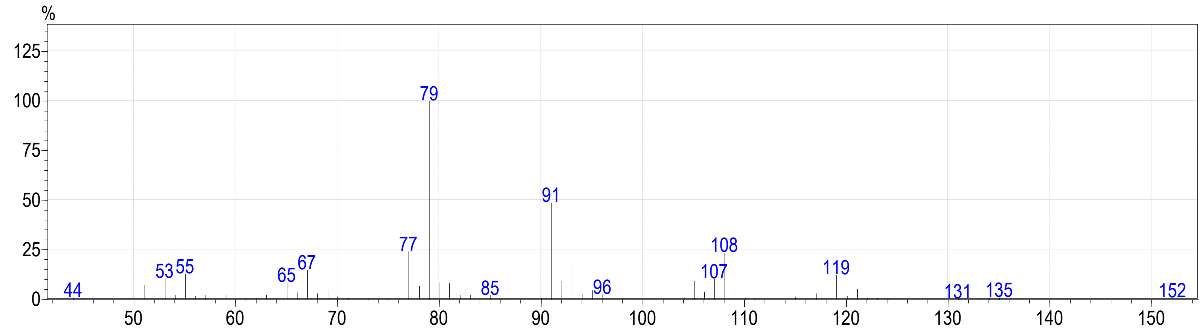


**Figure S11** Mass spectrum of **8**, calculated for $C_{10}H_{16}O$: 152; MS (70 eV, EI) observed m/z (%): 152 (0.64), 134 (1), 121 (5), 119 (12), 108 (22), 107 (8), 96 (2), 93 (17), 91 (49), 85 (2), 80 (8), 79 (100), 77 (24), 67 (15), 65 (9), 55 (13), 53 (11), 44 (1)

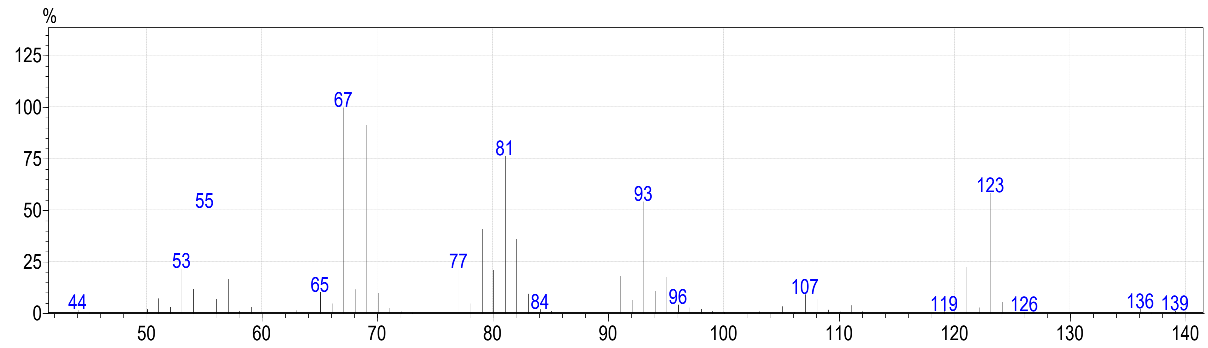


**Figure S12** Mass spectrum of **10**. Calculated for $C_{10}H_{18}O$: 154;  MS (70 eV, EI) observed m/z (%): 139 (1), 136 (2) ($M^{+\cdot}$-18), 126 (1), 123 (58), 119 (4), 107 (9), 96 (4), 93 (57), 91 (33), 84 (2), 82 (40), 81 (76), 79 (45), 77 (25), 69 (94), 67 (100), 65 (12), 55 (51), 53 (24), 44 (3).

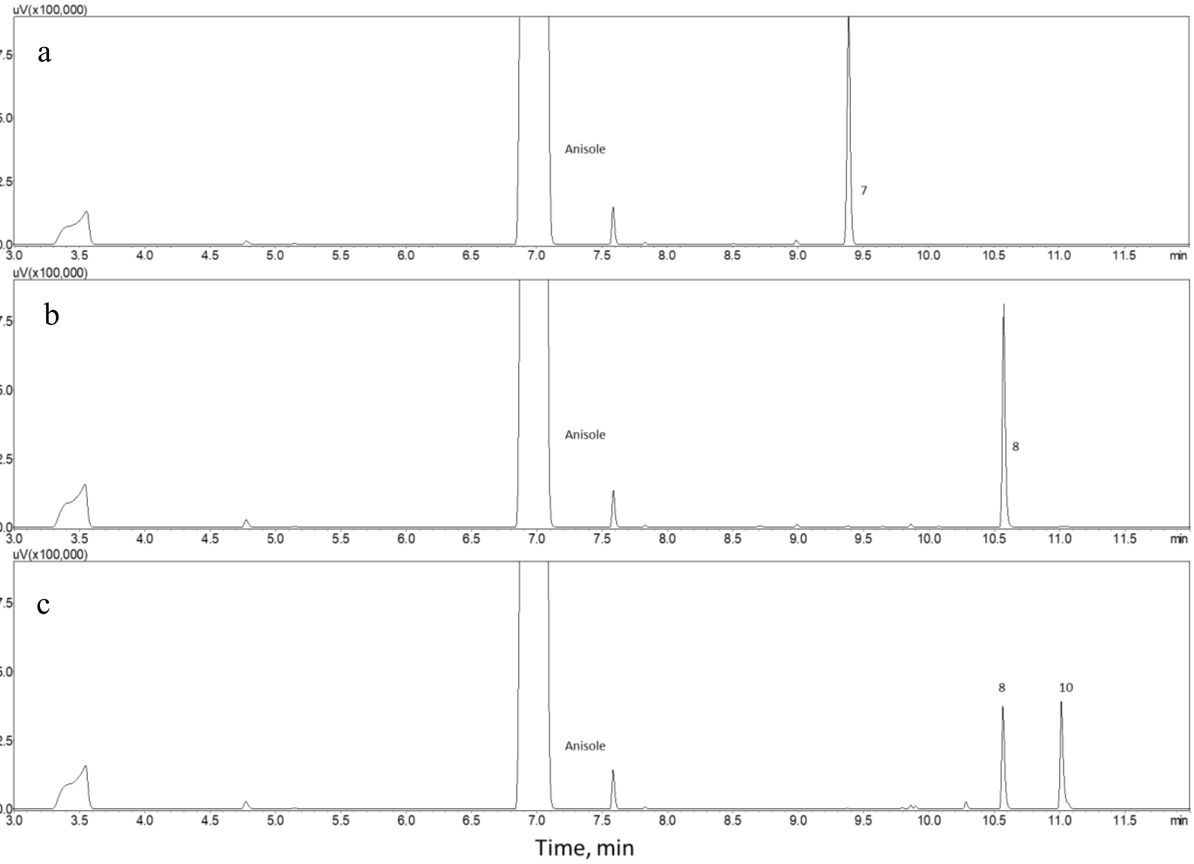


**Figure S13** Representative GC trace analysis of the transfer hydrogenation products of Myrtenal (**7**) under GC-FID conditions described in **S1** – Entry 1 of Table 2. Reaction Conditions: Myrtenal (0.133 mmol), **Ru-2** (1 mol%), Anisole (3 mL), EtOH (2.66 mmol), NaOMe (5 mol%); 30 °C. a) reaction time = 0; b) reaction time = 5 min. c) reaction time = 24 h, 80°C.

**Table S7** Areas of GC Analysis of the runs in Table 2.[a]

| Entry (time) | Internal standard | Peak (RT) | Peak (RT) | Peak (RT) | Peak (RT) |
|---|---|---|---|---|---|
| | Undecane (3.41) | **7** (9.389) | **8** (10.572) | **10** (11.014) | Others |
| 1 (0) | 1245221 | 1085824 | 0 | 0 | 0 |
| 1 (5 min) | 1441690 | 0 | 1248288 | 0 | 0 |
| 1 (24 h) [b] | 1484466 | 0 | 573739 | 654604 | 0 |
| 2 (5 min) | 1225693 | 1559744 | 0 | 0 | 0 |
| 2 (24 h) [b] | 1252772 | 0 | 1239976 | 208085 | 114570 |
| 3 (5 min) | 1487370 | 0 | 1148390 | 144442 | 0 |
| 3 (24 h) [b] | 1565505 | 0 | 0 | 1203401 | 218793 |
| 4 (5 min) | 1660833 | 23820 | 1154968 | 170199 | 0 |
| 4 (24 h) [b] | 1655473 | 0 | 399833 | 831460 | 53115 |
| 5 (5 min) | 1425019 | 0 | 1235954 | 159554 | 0 |
| 5 (24 h) [b] | 1478190 | 0 | 0 | 1367618 | 139331 |
| 6 (5 min) [c] | 1394963 | 9731 | 1409726 | 0 | 0 |
| 6 (24 h) [b], [c] | 1588046 | 0 | 346900 | 1109132 | 98004 |

[a]Reaction Conditions: Myrtenal (0.133 mmol), catalyst (1 mol%), Anisole (3 mL), EtOH (2.66 mmol), NaOMe (5 mol%); c.a. 5 min; c.a. 30 °C. [b] 80°C [c]Without base. RT = retention time

**Table S8** Areas of GC Analysis of the runs in Table 3.[a}

| Entry (time) | Internal standard | Peak (RT) | Peak (RT) | Peak (RT) |
|---|---|---|---|---|
| | Undecane (3.41) | **7** (9.389) | **8** (10.572) | **10** (11.014) |
| 1 (0) | 1245221 | 1085824 | 0 | 0 |
| 1 (5 min) | 1302438 | 1014638 | 49715 | 0 |
| 1 (1 h) | 1313902 | 566481 | 130027 | 0 |
| 2 (5 min) | 1448322 | 0 | 1114300 | 0 |
| 2 (1 h) | 1459559 | 0 | 1129260 | 0 |
| 3 (5 min) | 1421048 | 523272 | 498154 | 0 |
| 3 (1 h) | 1423672 | 98592 | 935878 | 0 |

[a]Reaction Conditions: Myrtenal (0,133 mmol), catalyst (0.1 mol%), Anisole (3 mL), EtOH (2.66 mmol), NaOMe (5 mol%); 1h, 35 °C. RT = retention time

**S6.3** Product characterization for the transfer hydrogenation of cinnamaldehyde (**11**)

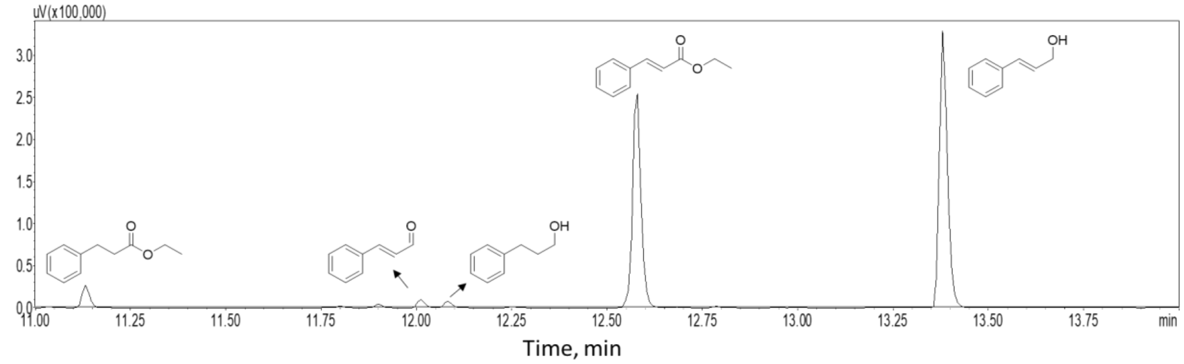


**Figure S14** Representative GC trace analysis of the transfer hydrogenation products of cinnamaldehyde (**11**), characterized by GC-MS.

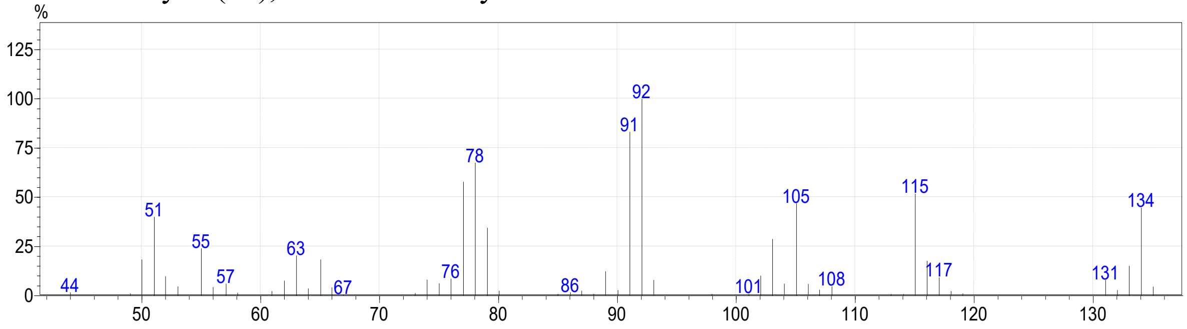


**Figure S15** Mass spectrum of **12.** Calculated for $C_9H_{10}O$: 134; MS (70 eV, EI) observed m/z (%): 135 (5), 134 (45), 133 (15), 132 (3), 131 (8), 119 (1), 118 (2), 117 (9), 116 (18), 115 (52), 114 (1), 113 (1), 108 (5), 107 (3), 106 (6), 105 (47), 104 (6), 103 (29), 102 (10), 101 (1), 97 (1), 92 (100), 93 (8), 91 (83), 90 (3), 89 (12), 88 (1), 87 (3), 86 (2), 85 (1), 80 (3), 79 (35), 78 (68), 77 (58), 76 (9), 75 (6), 74 (8), 73 (1), 67 (1), 66 (4), 64 (4), 65 (18), 63 (21), 62 (8), 60 (2), 58 (2), 57 (6), 56 (4), 55 (24), 53 (5), 52 (10), 51 (40), 50 (18), 49 (1), 44 (2)

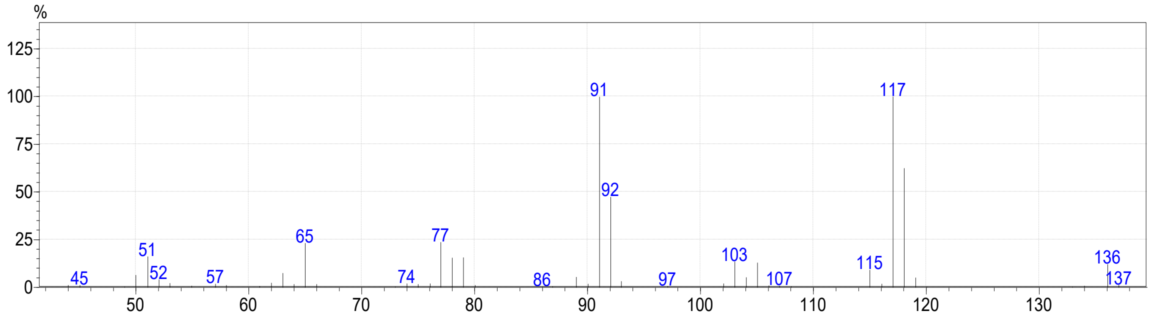


**Figure S16** Mass spectrum of **14**. Calculated for $C_9H_{12}O$: 136; MS (70 eV, EI) observed m/z (%): 137 (1), 136 (12), 134 (1), 133 (0), 119 (5), 118 (63), 117 (100),116 (2), 115 (9), 108 (1), 107 (1), 106 (1), 105 (13), 104 (5), 103 (14), 102 (2), 97 (1), 93 (3), 92 (48), 91 (99.80), 90 (2), 89 (5), 86 (1), 80 (1), 79 (16), 78 (16), 77 (24), 76 (2), 75 (2), 74 (2), 72 (0), 66 (2), 65 (23), 64 (2), 63 (8), 62 (2), 60 (1), 58 (1), 57 (2), 55 (1), 53 (2), 52 (4), 51 (16), 50 (7), 45 (1), 44 (1).

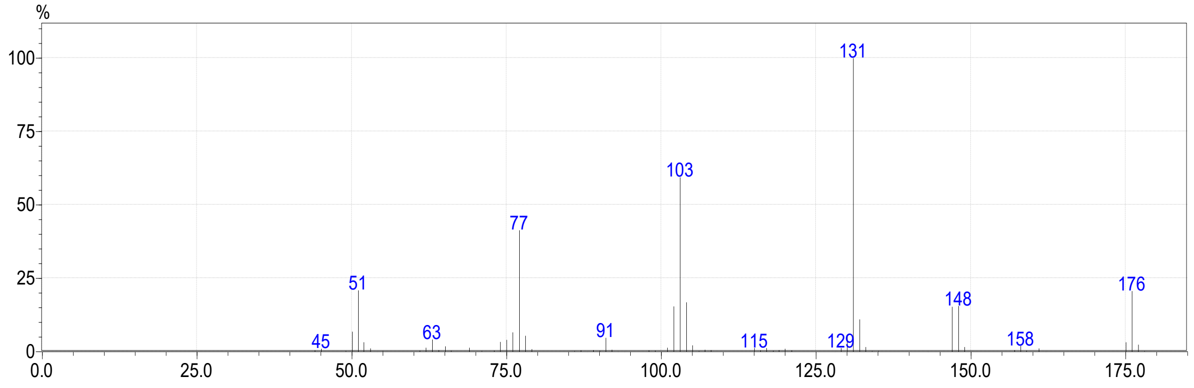


**Figure S17** Mass spectrum of **15**. Calculated for $C_{11}H_{12}O_2$: 176; MS (70 eV, EI) observed m/z (%): 178 (0.23), 177 (3), 176 (20), 175 (3), 131 (100), 161 (1), 158 (2), 157 (1), 149 (2), 148 (15), 147 (15), 133 (2), 132 (11), 130 (5), 129 (1), 121 (1), 120 (1), 119 (1), 117 (1), 116 (1), 115 (1), 108 (1), 107 (1), 105 (2), 104 (17), 103 (59), 102 (15), 101 (1), 98 (1), 92 (1), 91 (5), 89 (1), 87 (1), 79 (1), 78 (5), 77 (41), 76 (7), 75 (4), 74 (3), 69 (1), 65 (1.85), 64 (1), 63 (4), 62 (1), 53 (1), 52 (3), 51 (21), 50 (7), 45 (1), 44 (1).

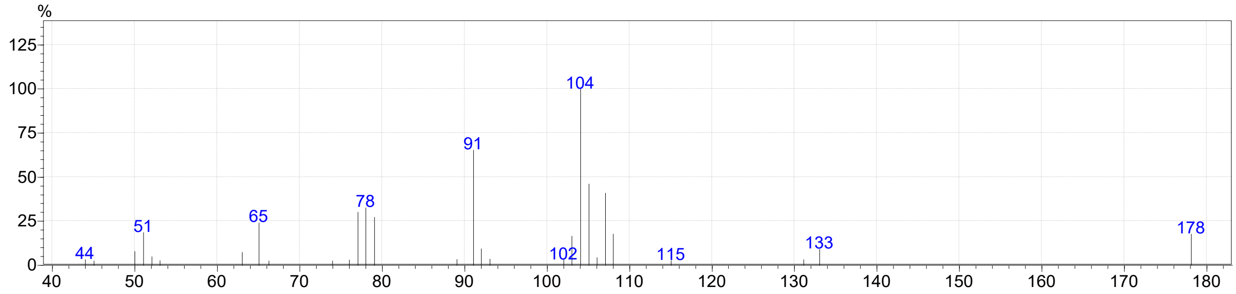


**Figure S18** Mass spectrum of **16.** Calculated for $C_{11}H_{12}O_2$: 178; MS (70 eV, EI) observed m/z (%): 178 (18), 133 (9), 131 (3), 115 (3), 108 (18), 107 (41), 106 (5), 105 (46), 104 (100), 103 (17), 102 (3), 93 (4), 92 (10), 91 (66), 89 (4), 79 (27), 78 (33), 77 (30), 76 (3), 73 (3), 66 (3), 65 (24), 63 (8), 53 (3), 52 (5), 51 (19), 50 (8), 45 (3), 44 (3).

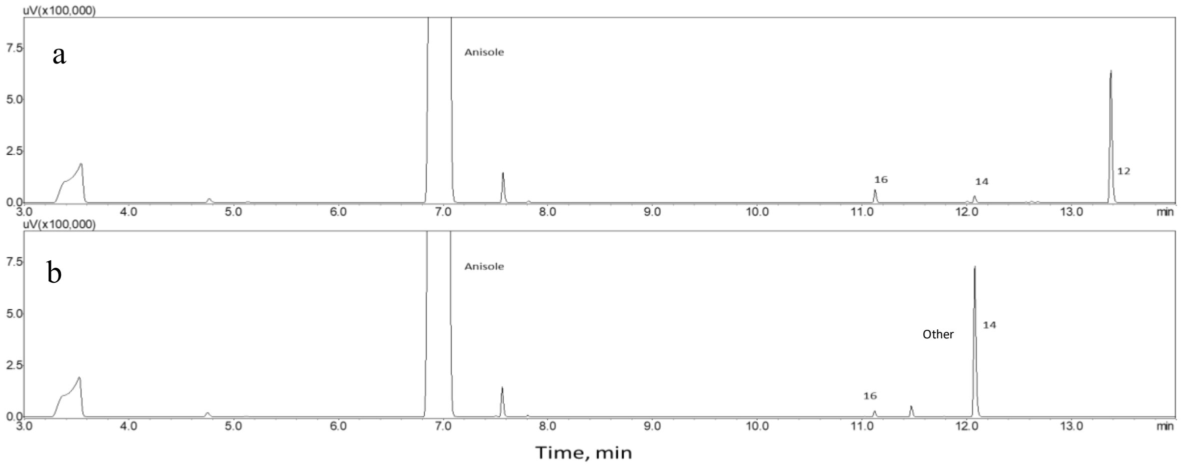


**Figure S19** Representative GC trace analysis of the transfer hydrogenation products of Cinnamaldehyde (**11**) under GC-FID conditions described in S1– Entry 3 of Table 4. Reaction Conditions: **11** (0.133 mmol), **Os-1** (1 mol%), Anisole (3 mL), EtOH (2.65 mmol), NaOMe (5 mol%); 80 °C. a) reaction time = 5 min, 30°C. b) reaction time = 24 h.

**Table S9** Areas of GC Analysis of the runs in Table 4. [a}

| Entry (time) | Internal standard | Peak (RT) | Peak (RT) | Peak (RT) | Peak (RT) | Peak (RT) | Others |
|---|---|---|---|---|---|---|---|
| | Undecane (3.41) | **11** (12.014) | **12** (13.384) | **14** (12.091) | 15 (12.557) | **16** (11.134) | |
| 1 (0) | 1335607 | 1158475 | 0 | 0 | 0 | 0 | 0 |
| 1 (5 min) [b] | 1254042 | 9320 | 425750 | 0 | 406025 | 0 | 0 |
| 1 (24 h) | 1210852 | 0 | 346718 | 130058 | 11242 | 312991 | 0 |
| 2 (5 min) [b] | 1318923 | 33022 | 349354 | 21852 | 293654 | 0 | 0 |
| 2 (24 h) | 1305604 | 0 | 101155 | 493417 | 0 | 31354 | 29660 |
| 3 (5 min) [b] | 1813437 | 0 | 1007769 | 40125 | 0 | 90008 | 0 |
| 3 (24 h) | 1816894 | 0 | 0 | 1098388 | 0 | 44883 | 0 |
| 4 (5 min) [b] | 1413491 | 0 | 872072 | 140956 | 0 | 152934 | 0 |
| 4 (24 h) | 1581101 | 0 | 0 | 1109911 | 0 | 105737 | 72891 |
| 5 (5 min) [b] | 1358952 | 0 | 908840 | 89164 | 0 | 131994 | 0 |
| 5 (24 h) | 1346362 | 0 | 0 | 1027633 | 0 | 54366 | 72243 |
| 6 (5 min) [b], [c] | 1315818 | 13624 | 743141 | 0 | 49127 | 0 | 0 |
| 6 (24 h) [c] | 1304522 | 0 | 66197 | 656356 | 0 | 93068 | 40305 |

[a]Reaction Conditions: **11** (0.133 mmol), catalyst (1 mol%), Anisole (3 mL), EtOH (2.65 mmol), NaOMe (5 mol%); 80 °C. [b]Time to quench the reaction at c.a. 5 min; c.a. 30 °C. [c]Without base.

**Table S10** Areas of GC Analysis of the runs in Table 5.[a]

| Entry (time) | Internal standard | Peak (RT) | Peak (RT) | Peak (RT) | Peak (RT) | Peak (RT) | Others |
|---|---|---|---|---|---|---|---|
| | Undecane (3.41) | **11** (12.014) | **12** (13.384) | **14** (12.091) | **15** (12.557) | **16** (11.134) | |
| 1 (0) | 1335607 | 1158475 | 0 | 0 | 0 | 0 | 0 |
| 1 (5 min) [b] | 1327755 | 712415 | 321023 | 0 | 30501 | 0 | 0 |
| 1 (24 h) | 1337975 | 0 | 1044152 | 0 | 50240 | 0 | 0 |
| 2 (5 min) [b] | 1337340 | 99605 | 888057 | 0 | 113076 | 0 | 0 |
| 2 (24 h) | 1349148 | 0 | 1001586 | 0 | 123444 | 0 | 0 |
| 3 (5 min) [b] | 1304949 | 476650 | 345903 | 0 | 42589 | 0 | 0 |
| 3 (24 h) | 1292747 | 0 | 845580 | 0 | 93865 | 0 | 0 |
| 4 (5 min) [b] [c] | 1321107 | 1145016 | 0 | 0 | 0 | 0 | 0 |
| 4 (24 h) [c] | 1308438 | 1143548 | 13437 | 0 | 0 | 0 | 0 |

[a]Reaction Conditions: Cinnamaldehyde **11** (0,133 mmol), catalyst (0.1 mol%); Anisole (3 mL), EtOH (2.65 mmol), NaOMe (5 mol%); 35 °C. [b] Time to quench the reaction at c.a. 5 min; c.a. 30 °C. [c]Without base.

## S7 References


[1] Spasyuk, D.; Gusev, D. Acceptorless dehydrogenative coupling of ethanol and hydrogenation of esters and imines. *Organometallics* **2012**, 31 (15), 5239–5242. DOI: 10.1021/om300670r

[2] Spasyuk, D.; Smith, S.; Gusev, D. G. Replacing phosphorus with sulfur for the efficient hydrogenation of esters, *Angewandte Chemie International Edition* **2013**, 52, 2538–2542. DOI: 10.1002/anie.201209218

[3] Spasyuk, D.; Vicent, C.; Gusev, D. G. Chemoselective hydrogenation of carbonyl compounds and acceptorless dehydrogenative coupling of alcohols, *Journal of the American Chemical Society* **2015**, 137 (11), 3743–3746. DOI: 10.1021/ja512389y
